# Starter Yeast Selection and Utilization for the Production of Fermented-Fruit-Derived Beverages: Methodologies and Recent Advances

**DOI:** 10.3390/foods15152683

**Published:** 2026-07-30

**Authors:** Giuseppe Romano, Francesco Loperfido, Valentina Petrelli, Pasquale Venerito, Aronne Galeotti, Lorenzo Palombi, Francesco Grieco, Maria Tufariello

**Affiliations:** 1 Institute of Sciences of Food Production-National Resarch Council (ISPA-CNR), Via Prov.le Lecce-Monteroni, 73100 Lecce, Italy; giuseppe.romano@cnr.it (G.R.); maria.tufariello@cnr.it (M.T.); 2 Istituto Tecnico Superiore, Settore Produzioni Agroalimentari, Fondazione ITS Academy AgriPuglia, 70010 Locorotondo, Italy; lope.francesco@gmail.com (F.L.); v.petrelli@itsagroalimentarepuglia.it (V.P.); aronne.galeotti@gmail.com (A.G.); 3 CRSFA Centro di Ricerca, Sperimentazione e Formazione in Agricoltura Basile-Caramia, Via Cisternino 281, 70010 Locorotondo, Italy; pasqualevenerito@crsfa.it; 4 Institute of Applied Physics “Nello Carrara”-National Research Council (IFAC-CNR), Via Madonna del Piano, 10, 50019 Sesto Fiorentino, Italy; l.palombi@ifac.cnr.it

**Keywords:** fruit wine, fermented beverage, yeast selection, biodiversity

## Abstract

The production of fermented beverages using different fruits has attracted increasing interest due to evolving consumer preferences (e.g., health consciousness and demand for low-alcohol products) and technological advances in the production process. Yeasts play a fundamental role in determining the sensory, chemical and functional properties of these beverages. This review aims to summarize the latest scientific publications on the selection and use of *Saccharomyces* and non-*Saccharomyces* starter yeasts for juice processing. It illustrates and discusses the most recent findings on isolation and characterization techniques, from phenotypic screening to sophisticated ‘omics’ approaches, which allow precise identification and engineering of yeast strains. The impact of various inoculation strategies, such as single-strain inoculation, sequential inoculation, and co-fermentation with different strains, is analyzed according to its effect on the quality and complexity of the final product. Recent advances in the application of genomics and metabolomics to re-evaluate fruit by-products highlight the importance of innovation in developing new, sustainable, diverse fermented fruit beverages.

## 1. The Production of Fermented Fruit-Based Beverages

Alcoholic fermentation is a metabolic process involving the conversion of simple carbohydrates into alcohol and carbon dioxide. Yeasts, particularly those belonging to the *Saccharomyces cerevisiae* species, play a key role in this process [1]. It is one of the oldest and most significant biotechnological applications, allowing not only the production of fermented beverages, but also a wide range of other products such as bread and cheese [1]. Selecting optimal yeast strains for each production process is essential for enhancing the sensory quality of the final product [2]. In addition, fermentation is an efficient and economical method of preserving perishable raw materials such as fruit juices, particularly in tropical regions where preventing fruit spoilage is a significant challenge [3]. Fermentation has been used for generations to preserve plant products, such as fruit, in the form of beverages, thus ensuring the safety of the final product for consumers [2]. In addition to the transformation of sugars into ethanol and carbon dioxide, yeast activity during fermentation produces hundreds of secondary metabolites that contribute to the aroma and flavour profile of the final product [1]. Searching for new starter yeast cultures is now recognized as a powerful way to improve the quality of the final product [4] and fermentation efficiency, as newly identified strains may possess advantageous traits such as enhanced tolerance to ethanol, osmotic, temperature, and pH stresses, resulting in improved fermentation productivity, as well as the ability to produce more desirable aroma and flavour compounds that enhance product quality [5].

Additionally, novel microbial strains can contribute to creating new functional foods and beverages that are enriched with antioxidants, probiotics, and other bioactive compounds [6]. This shifts the focus of the production of new fermented fruit beverages towards the active development of complex products with improved sensory and functional properties that are nutritionally valuable. This review was conducted as a narrative analysis of scientific literature concerning the selection and utilization of yeast starter cultures for fermented fruit-derived beverages. Relevant publications were identified through searches of major scientific databases, including Scopus, Web of Science, PubMed, and Google Scholar. The search was performed using combinations of keywords such as “fruit wine”, “fermented fruit beverages”, “yeast selection”, “starter cultures”, “*Saccharomyces*”, “non-*Saccharomyces*”, “co-fermentation”, “sequential inoculation”, “omics”, “genomics”, “metabolomics”, “fruit fermentation”, and “yeast biodiversity”. Priority was given to peer-reviewed articles published in English, with particular emphasis on recent publications and studies providing experimental evidence on yeast characterization, fermentation performance, aroma development, and starter culture applications. Additional references were identified through cross-checking the bibliographies of relevant publications. Studies not directly related to yeast-mediated fruit fermentations or lacking sufficient methodological information were excluded. The selected literature was used to provide a comprehensive overview of current methodologies, technological applications, and emerging trends in the field of fermented fruit beverages. The aim of this review is to provide an updated analysis of the most recent studies regarding the selection and use of *Saccharomyces* and non-*Saccharomyces* yeast starter cultures and to highlight progress in this area.

## 2. Fundamental Principles of Yeast Selection for Fruit Fermentation

### 2.1. Key Properties of Optimal Yeast Strains

The selection of the appropriate yeast strain to produce fruit-based fermented beverages is a critical step in the process, since the selected strain will greatly influence the organoleptic and nutritional characteristics of the final product. The microbial selection process requires knowledge of the intrinsic properties of the yeast strain as a function of the specific fruit juice used, the desired beverage style and the different fermentation process parameters (Table 1) [7].

Criteria important for selecting starter cultures include their ability to rapidly and completely consume sugars and synthesize fermentation by-products that contribute to a desirable aroma profile [12,13]. However, different yeast strains, particularly non-*Saccharomyces* species [3], exhibit different levels of tolerance with respect to the final alcohol concentration in the beverage (Table 1). Furthermore, the selected yeast strain must be able to withstand difficult environmental conditions that are often present during fermentation, such as temperature variations, high ethanol concentrations (>13% *v*/*v*), high osmotic pressure and acidity [3]. An important aspect of the microbial starter selection protocol is the characterization of strains that can positively influence the beverage’s organoleptic profile [8]. Yeast strains produce numerous by-products, particularly volatile esters, which significantly contribute to the complex flavour of fermented alcoholic beverages [14]. The aim of the selection process is therefore to increase the concentration of desirable compounds, such as those that impart fruity or floral notes, while avoiding the presence of undesirable compounds, such as acetic acid, hydrogen sulphide (H_2_S) and acetaldehyde [4]. In fact, excessive concentrations of acetaldehyde (over 100 mg/L) can have a negative impact on the flavour of the product [15], as can an excess of volatile acids and H_2_S, which can impart an unpleasant flavour [12]. Other practical considerations in yeast selection include technological parameters such as flocculation capacity and foam production, which influence the production process [12]. Furthermore, it is important to consider integrating specific nutrients for a given yeast strain, especially when fermenting juices obtained from fruit varieties that are naturally deficient in yeast nutrients and have a high sugar content, such as those with limited availability of Yeast Assimilable Nitrogen (YAN < 140 g/L) [12]. The selection criteria for yeast strains consist of a complex system of interconnected parameters. The resilience of a yeast strain to stress conditions such as high final ethanol concentrations and high acidity directly influences fermentation efficiency and the consequent ability to produce secondary metabolites. A robust, stress-tolerant strain dominates the fermentation process completely and vigorously, contributing to the formation of the aroma and organoleptic properties of the final product [3]. The resilience of a yeast strain to fermentation-associated stresses contributes to fermentation robustness and process reliability by reducing the risk of sluggish or stuck fermentations. However, stress tolerance alone should not be considered a predictor of sensory quality, which remains strongly dependent on strain-specific metabolic characteristics and fermentation conditions.

### 2.2. Saccharomyces cerevisiae: An Established Tool and Its Advantages

*S. cerevisiae* is the most common and widely available yeast species on the market. It is historically used for producing fermented foods and beverages. Its widespread use is justified by the well-established knowledge of its two fermentative properties, which enable operators to predict the standardized production of fermented foods with consistent characteristics [16]. As one of the earliest domesticated ascomycete fungi to be domesticated and studied, strains belonging to this species are used extensively in industry, significantly influencing the quality, flavor and aroma of fermented foods [17]. Fermentation by *S. cerevisiae* of various primary substrates offers several notable advantages, including high ethanol production, robust fermentation and a contribution to the aroma of the final product [18].

Duarte et al. [19] evaluated the effects of sixteen different strains of *S. cerevisiae* and *Saccharomyces bayanus* to produce raspberry fruit wine. The authors recommended one strain of *S. cerevisiae* for the fermentation of raspberry juice, which allowed the production of fruit wine with high concentrations of acetates, higher alcohols and ethyl esters, and with low acidity. Commercial *S. cerevisiae* yeast strains have also been evaluated to ferment pomegranate juice, thus obtaining wine with both high ethanol concentration and total anthocyanin content [20]. Recent studies have focused on deepening our understanding of *S. cerevisiae* capabilities, leading to the development of specialized strains tailored for specific industrial applications, including reduced ethanol production, enhanced aroma and flavour profiles, improved stress tolerance, and increased fermentation efficiency [15,21].

These starter strains are selected for targeted applications and possess particular metabolic properties that enhance aromas and reduce off-flavors in fermented beverages [22], as well as increasing tolerance to stress and enabling the production of low-alcohol beverages [23]. Numerous experimental studies and reviews have been published in which the authors have used *S. cerevisiae* strains as primary starters (either pure or mixed with strains of the same species) for fruit matrices other than grapes. Regarding apple juice, Sun and colleagues [24] carried out fermentations of apple juice using *S. cerevisiae* SC125 as a pure starter, achieving high GABA production, an improved aroma profile (esters and higher alcohols), and good sensory acceptability. However, the high GABA production observed in the SC125 strain is a strain-specific trait of interest for the development of functional beverages. Indeed, tailor acidity and mouthfeel the reported yield was obtained in the presence of added MSG, which may limit direct extrapolation of these results to conventional fermentations. A mass screening and subsequent selection of indigenous *S. cerevisiae* strains for apple juice fermentation and cider production highlighted strain-dependent differences in the organoleptic properties [14]. The fermentation conducted by Estela-Escalante et al. [25] on apple juice using *S. cerevisiae* via modulated aeration resulted in ethanol and byproducts (glycerol, higher alcohols, esters), with a direct impact on the sensory quality of the final product.

The use of selected strains of *S. cerevisiae* has been documented for the fermentation of juices from different pear varieties with *S. cerevisiae*, resulting in the characterization of the volatile compounds and the sensory profile of the resulting beverage [26]. Recently, Huang and colleagues conducted a metabolomic and transcriptomic analysis of the fermentation of pear juices and concentrates using *S. cerevisiae*, highlighting the yeast’s metabolic adaptability to the fermentation of the fruit matrix [27].

A recent study documented the fermentation of orange and blackcurrant juice using *S. cerevisiae* var. *boulardii* as a starter culture [28]. This starter culture promoted good preservation of polyphenols, controlled alcohol production, and a satisfactory sensory profile. Furthermore, the fermentation of longan juice with *S. cerevisiae* var. *bayanus* was studied by Trinh et al. [29], demonstrating an improvement in the organoleptic profile compared to that observed in the same beverage obtained through spontaneous fermentation. Taken together, the above evidence indicates a move towards developing novel fermentation procedures, in which this traditionally used species is transformed into a highly modifiable tool. This will enable producers to customize the characteristics of the beverages produced, ensuring control of the fermentation process and responding to current production issues, such as climate change, and the demand of consumers for natural products with health benefits.

### 2.3. The Non-Saccharomyces Yeasts: Diversity and Emerging Roles

Non-*Saccharomyces* yeasts have undergone a significant re-evaluation in recent years. Historically, within the context of wine fermentation, non-*Saccharomyces* yeasts were frequently regarded as undesirable microorganisms or potential spoilage agents because of their association with specific fermentation defects. However, their role has been substantially re-evaluated in recent decades, and many species are now recognized as important contributors to fermentation performance, aroma complexity, analytical composition, and product diversification [30]. In contrast, several typical fermented beverages produced in different regions of the world have long relied on diverse non-*Saccharomyces* yeasts and mixed microbial communities, highlighting the context-dependent nature of their technological relevance. Their negative perception has diminished as numerous studies have demonstrated their crucial role in determining the sensory quality, as well as enhancing the analytical composition and aroma profile, of fermented beverages. This re-evaluation stems from their ability to produce valuable compounds that contribute to the final product’s quality, offering several distinct advantages in beverage production. These yeasts exhibit remarkable diversity, encompassing over 20 genera. Common species that are often isolated in spontaneous fermentations include *Hanseniaspora uvarum*, *Metschnikowia pulcherrima*, *Starmerella bacillaris*, *Pichia terricola*, *Torulaspora delbrueckii*, *Pichia kudriavzevii* and *Hanseniaspora guilliermondii* (Table 1).

The contributions of non-*Saccharomyces* yeasts are highly species- and even strain-specific, emphasizing the critical importance of judicious strain selection [9]. Their diverse metabolic activities enable them to enhance aroma and flavor, improve chemical composition and ensure color stability and biocontrol capabilities [9,23]. The potential of non-*Saccharomyces* yeasts is being increasingly explored as a means of mitigating the challenges posed by climate change, such as higher sugar content in must, and of meeting evolving market demands for new and differentiated products [31].

Chen et al. [32] investigated the fermentative performance of strains of *T. delbrueckii*, *Williopsis saturnus*, and *Kluyveromyces lactis* employed for the production of lychee wine. The *T. delbrueckii* showed both the highest growth rate and level of ethanol, whereas *K. lactis* and *W. saturnus* produced a high amount of the undesired ethyl acetate which was considered detrimental to the final product quality. Indeed, the *T. delbrueckii* strain synthesized high levels of positive aroma-character compounds, it being suitable as candidate starter for wine fermentation. Several non-*Saccharomyces* yeast species were evaluated for their fermentation properties and the production of pleasant fruity aromas in cider [30]. A total of 99 yeasts were screened for aroma production using a simple olfactory plate assay. Strains belonging to *Galactomyces geotrichum*, *Kazachstania zonata*, *Kluyveromyces lactis*, *Lindnera meyerae*, *P. kluyveri*, *Starmera caribae*, *Yarrowia lipolytica* and *Saccharomycodes ludwigii* species showed the potential to produce pleasant aroma compounds during apple juice fermentation. A recent study by Liu and coworkers [33] aimed at evaluating the quality of fig wines co-fermented with three strains of *P. fermentans*, *H. uvarum* and *Wickeramomyces anomalus*. When compared to the fig juice fermented with the control *S. cerevisiae* starter, the fig wines produced by the non-*Saccharomyces* showed higher contents of aroma compounds, mono-phenols and organic acids. Wei and colleagues [34] carried out simultaneous fermentation of apple juice of non-*Saccharomyces* yeasts to produce cider with enhanced flavor complexity. The authors demonstrated the potential effect of *P. kluyveri* and *Hanseniaspora vineae* species on the sensorial properties of cider when used in mixed fermentations with *H. uvarum* or *T. quercuum*.

## 3. Methodologies for Isolation, Identification, and Characterization of Starter Yeasts

### 3.1. Isolation from Fruit Matrices and Preliminary Characterization

The first and most important step in identifying novel yeast strains with biotechnological potential is to isolate them from various natural sources, particularly different types of fruit (see Table 2). These sources include Amazonian native fruits, which harbor rich microbial diversity. The isolation process typically begins with careful sampling. Fruits are collected at the ripe stage and in good condition and are then transported to the laboratory in refrigerated and sterile bags to maintain microbial viability [35,36]. Samples can be taken from different parts of the fruit, such as whole fruits, stalks or skins [37]. After collecting, the preparation process involves pooling fruits of the same species and washing them aseptically with sterile water. Serial decimal dilutions of the wash water or fruit pulp are then prepared in liquid growth media and then sown, by spatulation (Spread Plate), in plates set up with a solid culture medium. Finally, colony selection and purification are performed. Individual colonies are chosen based on distinct morphological characteristics, including form, size, color, margin and elevation [35]. Phenotypic characterization is a vital component of yeast selection, serving as a rapid and informative initial screening tool to understand the functional properties and potential industrial applicability of the isolated yeast strain [8]. This preliminary step includes macroscopic and microscopic observation, as well as physiological tests such as sugar fermentation, osmotic, temperature, pH, NaCl and ethanol tolerance, and SO_2_ resistance.

Although seemingly basic, phenotypic profiling offers a rapid and comprehensive initial assessment of a yeast strain’s suitability for a specific fermentation process [37]. These tests provide invaluable insights into a strain’s metabolic capabilities, resilience to environmental stresses and propensity for producing desirable or undesirable metabolites (such as acetic acid and H_2_S). This multifaceted approach enables researchers to narrow down a large pool of candidates, identifying promising strains and ruling out unsuitable ones early in the selection process, before committing to more resource-intensive molecular analyses. Consequently, phenotypic characterization remains a cornerstone of yeast selection, providing practical, actionable data for initial screening and guiding subsequent, more detailed investigations in a cost-effective manner [37,39,40].

### 3.2. Molecular Identification and Characterization Techniques

Molecular methods are important for accurately and precisely identifying yeast species and strains, particularly after initial phenotypic screening has narrowed down the potential candidates [13]. The process typically begins with DNA extraction, followed by PCR-based methods that are widely used for species- and strain-level differentiation: These include Rep-PCR (Repetitive Extragenic Palindromic Sequences PCR) and RFLP-PCR (Restriction Fragment Length Polymorphism PCR) of the 5.8S-ITS region, as well as sequencing of the Internal Transcribed Spacers (ITS) and D1/D2 domains of the 26S rRNA gene [35]. Sequencing and phylogenetic analysis provide definitive identification and insights into evolutionary relationships through database comparison.

Recent advancements have led to the integration of genomic and ‘omics’ approaches, transforming yeast characterization from identification to predictive functional analysis by phylogenomic and functional annotation. This enables the deciphering of complex metabolic networks related to fermentation capacity and the modulation of flavor and aroma profiles, known as the ‘fermentome’ and ‘flavourome’ [31]. Recently, omics technologies, including genomics, proteomics and metabolomics, have enabled the identification of specific genes and enzymes directly involved in flavor and aroma production. These technologies also facilitate the detection of increased levels of bioactive compounds in fermented products, offering a comprehensive understanding of yeast metabolic contributions [38].

These modern techniques go beyond mere taxonomic identification, providing an understanding of the functional potential of yeast strains at a deep genetic level [31,36,41]. Identifying a ‘fermentome’ and ‘flavorome’ means researchers can predict a strain’s fermentative capabilities and specific organoleptic contributions based on its genetic blueprint rather than relying on extensive empirical trials. This fundamentally changes the paradigm from reactive screening to proactive, genome-informed selection. This capability enables more targeted and efficient strain selection, potentially reducing the time and costs associated with product development, and facilitating the precise engineering or selection of yeasts with specific desired characteristics for fermented fruit beverages [31]. However, despite their considerable value for strain characterization and selection, the predictive power of genomics and other omics approaches remains subject to important limitations. In yeasts, genotype–phenotype relationships are often complex and non-linear, being influenced not only by the presence of specific genes but also by genetic background, regulatory mechanisms, epistatic interactions, and gene–environment interactions [42]. The phenotypic expression of fermentation-relevant traits is strongly affected by environmental variables such as temperature, nutrient composition, oxygen availability, and fermentation management practices. Consequently, genetic and molecular signatures associated with fermentation performance or aroma production do not necessarily translate into equivalent phenotypic outcomes under different fermentation conditions. Therefore, omics-based analyses should be regarded as complementary tools that support strain selection and functional interpretation, while experimental validation through phenotypic screening and fermentation trials remain essential [43].

### 3.3. Advanced Screening Protocols for Desirable Traits

Beyond fundamental evaluation, advanced screening protocols are systematically employed to improve the selection of yeast strains and achieve highly specific characteristics in fruit wines and other fermented fruit beverages [37]. Although phenotypic methods such as Durham tube assays, physiological characterization, and enzymatic activity tests provide valuable preliminary information on yeast metabolic capabilities, they do not reveal the underlying genetic determinants of these traits nor reliably predict strain performance under industrial fermentation conditions, where complex interactions among environmental factors may substantially influence yeast behavior.

A multi-stage evaluation process is commonly used, integrating preliminary screening, secondary evaluation and final-stage assessments to ensure comprehensive evaluation [40]. Preliminary screening measures the physical and chemical parameters of the fermented samples, such as total sugar, total acidity and alcohol content. The secondary evaluation then uses rapid methods to identify strains with robust fermentation capabilities, such as weight-based analysis and initial aroma analysis using gas chromatography–flame ionization detection (GC–FID). Sensory assessments may also be conducted at this stage to evaluate initial quality attributes. The final stage consists of an in-depth analysis of yeast tolerance to critical fermentation inhibitors such as ethanol and sulfur dioxide (SO_2_), and a detailed analysis of the volatile compounds of wine is performed using advanced techniques such as gas chromatography–mass spectrometry (GC–MS) [39].

Response surface methodology (RSM) is an advanced statistical tool that has recently been adopted to optimize fermentation conditions. RSM enables systematic investigation of multiple factors, providing a structured approach to identifying optimal process parameters and leading to enhanced, predictable outcomes [37]. Sensory analysis remains a critical component throughout the screening process. Methods such as hedonic sensory analysis and the check-all-that-apply (CATA) method are employed to evaluate the effects of different yeast strains and co-culturing strategies on the beverage’s overall quality and consumer acceptance [37]. Finally, small-scale fermentation trials are conducted with the selected strains using actual fruit juice, enabling the practical validation of laboratory findings under conditions closely mimicking industrial production [8].

**Figure 1:**
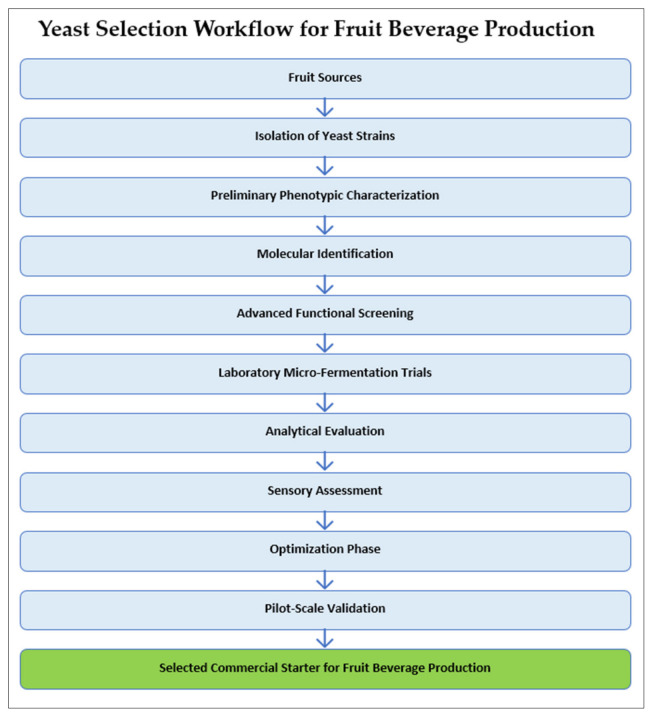
Flowchart showing the selection procedure of yeast starter cultures for fruit beverage production, from strain isolation and characterization to fermentation trials, optimization, pilot-scale validation, and final commercial starter selection.

Integrating multi-stage screening protocols, advanced analytical techniques such as GC-MS and GC-FID, and sophisticated statistical optimization tools like RSM represent a significant evolution in yeast selection. This transition moves the process away from the trial-and-error approaches toward a more systematic and predictive strategy. The overall workflow for yeast starter selection, from isolation to pilot-scale validation and commercial application, is summarized in Figure 1.

## 4. Strategies for Starter Yeast Utilization in Fruit Fermentation

### 4.1. Single-Strain Inoculation: Advantages and Limitations

The choice of inoculation strategy has a significant influence on the final characteristics of fermented fruit beverages. Modern production employs various approaches, each with its own advantages and limitations (see Table 3). Single-strain inoculation involves introducing a single, carefully selected yeast strain to initiate and control the entire fermentation process [44]. This method has traditionally been the standard in industrial settings due to its inherent advantages.

The primary benefit of single-strain inoculation is the predictability and consistency it offers. When a thoroughly characterized strain is used, its fermentative activity, ethanol production and general metabolic outputs are well established, ensuring uniform and predictable product quality [1]. This predictability makes process control and quality management easier for producers that aim for consistent product batches.

However, one problem with monoculture is that they often have less flavor complexity than fermentation strategies involving multiple yeast species [44]. For example, studies on durian wine have shown that using just one type of yeast (*S. bayanus* or *T. delbrueckii*) produced less acetyl esters and more alcohol compared to using two types of yeast together or adding the yeast in a certain order [50]. This suggests that while a single strain can efficiently convert sugars to ethanol, it may not have all the necessary enzymes or metabolic pathways to generate the full range of aroma compounds that contribute to a complex sensory profile.

When choosing yeast, it is important to balance how easy it is to predict and how complex it is. Single-strain inoculation gives more control and consistency, but it also limits how deep and rich the flavor of the wine can be, because it stops the different types of yeast from interacting with each other [44]. The above inherent limitation of single-strain inoculation is a key reason why mixed-culture strategies are growing in popularity. This is because producers want to make their products more complex and give them unique organoleptic characteristics.

### 4.2. Co-Fermentation: Synergies to Enhance Complexity

Co-fermentation involves the simultaneous inoculation of two or more yeast starter cultures into fruit must [51]. This approach primarily aims to exploit the complementary metabolic properties of different yeasts to enhance fermentation performance and increase the chemical and sensory complexity of the final beverage. These effects are often difficult to achieve using single-strain fermentation. The effect of mixed yeast cultures on ethanol production depends strongly on the yeast species involved, inoculation strategy, and fermentation conditions. For example, co-fermentation of *S. bayanus* and *T. delbrueckii* in durian must resulted in an ethanol content of approximately 6.2%, compared with 5.3–5.8% obtained in the corresponding monoculture fermentations, suggesting a positive interaction between the two species under those specific conditions [44]. However, this outcome is not universal, as numerous studies have shown that mixed fermentations involving non-*Saccharomyces* yeasts can also reduce ethanol production by redirecting carbon flux toward biomass formation, respiration, glycerol synthesis, or other metabolites. Consequently, the effect of mixed cultures on ethanol yield is highly strain-dependent and must be evaluated on a case-by-case basis [9,52,53,54].

Beyond ethanol production, co-fermentation often increases the production of volatile aroma compounds, such as acetate esters and higher alcohols (e.g., isoamyl alcohol and 2-phenylethyl alcohol), which are associated with fruity and floral sensory notes [44]. In addition, mixed cultures have been reported to increase the concentrations of terpenes (e.g., α-terpineol and linalool) and volatile thiols (e.g., 3-mercaptohexanol and 3-mercaptohexyl acetate), which contribute to tropical fruit-like aromas [9]. In some cases, co-fermentation can also lead to the biosynthesis of volatile compounds that are absent in monocultures, such as thioesters in durian wine, suggesting modifications of metabolic pathways arising from yeast–yeast interactions [44]. Non-*Saccharomyces* yeasts can also influence the acid profile of fermented beverages. For example, species such as *T. delbrueckii* and *M. pulcherrima* have been reported to modulate the composition of organic acids, often resulting in reduced perceived acidity [9]. In particular, *Lachancea thermotolerans* is known for its ability to produce L-lactic acid during fermentation, thereby modifying the balance between malic and lactic acids and offering a means to tailor acidity and mouthfeel [9]. Despite its advantages, co-fermentation poses challenges in terms of the predictability and reproducibility of fermentation outcomes. Interactions among different yeast species are often complex and nonlinear, involving competition for nutrients, modulation of metabolic pathways, and interspecies signaling. Consequently, fermentation performance and sensory attributes cannot always be inferred from monoculture behavior. The complexity of mixed fermentations is also linked to specific biological interactions among yeast species. For example, cross-feeding phenomena may occur when metabolites produced by one microorganism, such as amino acids, vitamins, or intermediate fermentation products, are utilized by another species, thereby influencing growth dynamics and metabolite production. In addition, enzymatic complementarity between different yeasts can enhance the transformation of grape or fruit precursors into aroma-active compounds [55]. Non-*Saccharomyces* species often exhibit higher activities of enzymes such as β-glucosidases, esterases, or carbon–sulfur lyases than *S. cerevisiae*, promoting the release of terpenes, volatile thiol and other desirable aroma compounds. These cooperative interactions may contribute to enhanced complexity and distinct sensory profiles frequently observed in mixed-culture fermentations [56]. For this reason, preliminary small-scale trials are typically required to evaluate microbial compatibility and optimize inoculation strategies prior to industrial application [57].

It should be noted, however, that increased ethanol production should not be interpreted as a universal indicator of superior fermentation performance. Historically, maximizing ethanol yield was considered a major technological objective because it reflected efficient sugar conversion and enhanced microbiological stability. In contrast, contemporary research increasingly recognizes that high alcohol concentrations may negatively affect beverage balance, aroma perception, drinkability, and consumer acceptance. Moreover, growing consumer demand for low- and moderate-alcohol beverages has stimulated the development of alternative fermentation strategies aimed at limiting ethanol accumulation while preserving sensory quality [1].

In this context, several non-*Saccharomyces* species have attracted considerable interest due to their ability to divert carbon flux toward metabolites such as glycerol, organic acids, and other compounds rather than ethanol. Mixed and sequential fermentations involving species such as *M. pulcherrima*, *T. delbrueckii*, *L. thermotolerans*, and *S. bacillaris* have been investigated not only to enhance sensorial complexity but also as potential tools for reducing the final ethanol content of fermented beverages. Consequently, the success of a fermentation strategy should be evaluated using a broader set of criteria that includes sensory quality, aroma complexity, balance, consumer acceptance, and product typicity, rather than ethanol concentration alone [58,59].

Therefore, the optimal fermentation outcome is not necessarily the one that produces the highest alcohol content, but rather the one that best aligns with the desired technological and sensory characteristics of the final beverage [60].

Using mixed yeast cultures often results in synergistic interactions, leading to increased flavor complexity and higher sensory quality scores than single-strain fermentations [44]. Co-fermentation has also been shown to influence chromatic characteristics, including color intensity and stability, particularly in wines and other phenolic-rich fermented beverages. These effects are linked to the use of mixed inocula, as different yeast species can differentially adsorb anthocyanins onto their cell walls, modify phenolic extraction, and produce metabolites such as pyruvic acid and acetaldehyde that promote the formation of stable pyranoanthocyanins. Furthermore, interactions between *Saccharomyces* and non-*Saccharomyces* yeasts during co-inoculation or sequential inoculation may alter the anthocyanin profile and enhance color retention compared with monoculture fermentations. However, these effects are highly strain-dependent and may vary according to the inoculation strategy and fermentation conditions [61,62].

Lee et al. [63] have studied the formation of volatile compounds after fermentation of papaya juice by a mixed culture of *S. cerevisiae* and *W. saturnus*, comprising alcohols, aldehydes, esters and fatty acids. Notably, the mixed culture enhanced the formation of acetate esters such as 2-phenylethyl acetate, associated with rose, honey, and floral notes, and ethyl esters including ethyl octanoate, ethyl hexanoate, and ethyl decanoate, which contribute fruity, apple-, banana-, and floral-like aromas. Higher alcohols such as 2-phenylethanol, characterized by rose and honey descriptors, were also present at elevated levels. The authors demonstrated that the fermentation with this mixed starter culture resulted in the development of more complex aroma profile compounds and higher ethanol level than those using single yeasts.

The selection of fermentative starter culture for pear wine production has shown that a mixed starter inoculum containing *P. fermentans* and *S. cerevisiae* could be used to achieve high-quality fermented beverages from pear juice [64]. The authors demonstrated that a mixed culture of above yeast species enhanced both ethanol production (8.37% *v*/*v*) and aroma complexity in prickly pear wine through synergistic yeast interactions. In contrast, Zhang et al. [26] focused on low-alcohol pear beverages and showed that non-Saccharomyces yeasts produced lower ethanol levels (1.0–3.3% *v*/*v*) but increased aroma complexity, particularly through acetate ester formation.

Li and coworkers [47] successfully optimized the application of a mixed culture of *S. cerevisiae* and *W. saturnus* var. *mrakii* for the fermentation of mango juice, whereas the use of a mixed inoculum composed of strains of *Pichia fabianii*, *Saccharomycopsis fibuligera*, *P. kudriavzevii* and *S. cerevisiae* for the fermentation of masau fruit pulp enhanced the aroma profile of the resulting wines.

A mixture of non-*Saccharomyces* strains (*T. delbrueckii* and *M. pulcherrima*) and *S. cerevisiae* has been applied to produce cherry wines [65]. The addition of *M. pulcherrima* notably increased the concentration of higher alcohols, esters, acids and terpenes, whereas *T. delbrueckii* enhanced the presence of fruity esters and higher alcohols.

The group of Satora and coworkers [66] has assessed the effect of a mixed *Wickerhamomyces anomalus*/*S. cerevisiae* starter on the fermentation process of apple juice and chemical composition of the obtained cider, thus concluding that the mixed culture positively influenced the chemical and organoleptic profiles of apple wines. These results were confirmed by an analogous investigation carried out by Ye et al. [67].

However, Satora et al. [68] showed conflicting evidence regarding the use of *Schizosaccharomyces pombe* as a mixed starter culture with *S. cerevisiae* for apple juice fermentation. In fact, the cider produced with mixed yeast cultures yielded higher concentrations of ethanol and volatile esters compared to that produced with the respective pure cultures. These findings support the hypothesis that co-fermentation activates distinct metabolic interactions or enhances pathway fluxes through cooperative effects between species [9]. Sequential inoculation is a fermentation strategy where the first strain is added and left to ferment for a specific time, and then a second strain is added [9]. This method makes the most of the different metabolic activities and strengths of each yeast at different stages of the fermentation process, which are all carefully controlled.

This method can sometimes lead to even higher levels of ethanol production than co-fermentation. For example, adding *T. delbrueckii* and *S. bayanus* to durian wine one after the other led to a remarkable 7.7% *v*/*v* ethanol, which is more than that produced by either type of yeast alone or by both types together. Sequential inoculation is a very good way to control the bouquet precisely. It can lead to higher amounts of several compounds, especially ethyl esters and acetate esters, which add fruity and creamy flavours to the final product [44].

For example, sequential inoculation of *S. cerevisiae* following *W. saturnus* has been shown to improve the bouquet of mango wine, enhancing the production of desirable volatile compounds such as ethyl hexanoate, ethyl decanoate, ethyl octanoate, and various acetate esters. The success of this approach depends on careful optimization of both the inoculation ratio and its timing, as these parameters strongly influence metabolic interactions between yeast species. Inadequate control may lead to the excessive accumulation of compounds such as ethyl acetate, which can negatively affect product quality at high concentrations [44].

Co-fermentation involves the simultaneous interaction of multiple yeast species, leading to either competitive or synergistic effects. In contrast, sequential inoculation introduces a temporal component to yeast activity, allowing the targeted exploitation of the specific metabolic capabilities of each strain at different stages of fermentation. For instance, one yeast may modify the fermentation environment or produce precursor metabolites that are subsequently transformed by a second yeast strain. This temporal control enables fine-tuning of both the chemical composition and sensory attributes of the final product.

The strategic use of inoculation protocols, involving the addition of different yeast strains in a defined order, represents an effective tool for modulating fermentation performance and enhancing product complexity. This approach can overcome limitations associated with single-strain fermentations or uncontrolled co-fermentations, resulting in improved process control and more desirable flavor profiles.

## 5. Impact of Yeast Selection on Fermented Fruit Beverage Characteristics

### 5.1. Alcohol Yield and Fermentation Efficiency

Choosing the right kind of yeast is very important if you want to get the best possible alcohol yield from your fermented drinks. Different types of yeast can handle different levels of alcohol, which decides how much alcohol is in the final product [2].

Strains of *S. cerevisiae* are known for producing the highest concentrations of ethanol during fruit juice fermentation, they are like those found in wine [1]. It is interesting to note that modern fermentation methods, such as co-fermentation and sequential inoculation, have shown that it is possible to produce even more ethanol than monocultures. For example, when making durian wine, the amount of ethanol produced was 5.3–5.8% by volume when the grapes were fermented on their own. This increased to 6.2% by volume when the grapes were fermented together, and even more, to 7.7% by volume, when the grapes were fermented in sequence [44]. This shows that if different types of yeast work well together and are active at the right times, it can make the fermentation process more efficient.

However, recent research is also aiming to develop *S. cerevisiae* strains that can make less ethanol. This is because consumers are nowadays preferring low-alcohol drinks and wanting new and different low-alcohol products [1]. These strains are often created using methods like adaptive laboratory evolution and hybridization, which can lead to higher glycerol production, which is good for the mouthfeel [4].

The evolution of yeast selection has progressed beyond the mere maximization of alcohol yield to a more sophisticated approach of tailoring alcohol content to align with specific market trends. Recent studies and industrial practices have demonstrated an increasing focus on the deliberate selection and engineering of yeasts that are specifically engineered to reduce ethanol production. This shift can be considered a direct response to new consumer demands for “low-alcohol wines” and other innovative products [4]. This signifies a strategic pivot from a singular focus on ethanol yield to a diversified fermentation approach driven by specific market segments and the growing health-conscious consumer base. Consequently, the field of yeast research has become highly responsive to commercial demands, enabling precise control over alcohol content to cater to a broader range of preferences.

Increasing temperature is a key environmental factor influencing fruit composition and has important consequences for fermentation processes, particularly in fruit wine production. Elevated temperatures accelerate fruit ripening and enhance sugar accumulation (typically measured as °Brix) by promoting photosynthetic activity and metabolic fluxes toward carbohydrate storage. This effect has been widely documented under climate change scenarios, where rising temperatures have led to consistently higher sugar concentrations in grapes and other fruits at harvest [69].

As a direct consequence, higher sugar concentrations in the raw material translate into increased ethanol production during alcoholic fermentation, since yeast converts fermentable sugars into ethanol and carbon dioxide. Indeed, global warming has been associated with a measurable rise in alcohol levels of wines, primarily driven by enhanced sugar accumulation during ripening. Experimental studies further confirm that elevated temperatures not only increase °Brix but also result in wines with higher alcohol content [70].

This phenomenon represents a major challenge for modern fruit wine technology. Excessive alcohol levels can negatively affect sensory balance, microbial stability, and fermentation kinetics, while also requiring adjustments in both vineyard practices and fermentation strategies. Consequently, understanding and managing the temperature–sugar–ethanol relationship has become a critical aspect in the design and optimization of contemporary fruit fermentation processes [71].

The development of low-alcohol fermented beverages has stimulated the exploration of several strategies for obtaining yeast strains with reduced ethanol-producing capacity. These approaches include the selection of naturally occurring strains, Adaptive Laboratory Evolution (ALE), interspecific hybridization, and genetic engineering. ALE allows the selection of variants with modified carbon fluxes and altered fermentation efficiencies through prolonged cultivation under defined selective conditions, whereas hybridization combines desirable traits from different yeast species without the direct introduction of foreign DNA. More recently, metabolic engineering approaches have targeted pathways involved in central carbon metabolism with the aim of redirecting sugar utilization from ethanol production toward alternative metabolites such as glycerol or organic acids [72,73]. Reducing ethanol concentration may have important consequences for sensory quality. Ethanol contributes directly to sweetness, viscosity, body, and mouthfeel, while also affecting the volatility and perception of aroma compounds. Consequently, excessive alcohol reduction may result in beverages perceived as thinner, less balanced, and less aintense. For this reason, current strain development strategies seek to achieve moderate reductions in ethanol content while preserving desirable sensory characteristics through the production of glycerol, esters, higher alcohols, and other aroma-active metabolites [59]. Although genetically modified yeasts have shown considerable potential for controlling ethanol yield and improving fermentation performance, their industrial application remains subject to regulatory and societal constraints. Regulatory frameworks differ substantially among countries. In the European Union, genetically modified microorganisms intended for food production are subject to strict authorization procedures and labelling requirements under GMO legislation. In contrast, some non-GM breeding strategies, such as adaptive evolution and hybridization, generally face fewer regulatory barriers and are more readily accepted by consumers and producers. While genetically engineered yeasts have been successfully evaluated in wine and other beverage fermentations at the experimental level, commercial applications remain relatively limited, reflecting both regulatory restrictions and market acceptance considerations [73,74].

### 5.2. Modulation of Aroma and Flavor Profiles

Understanding the impact of volatile organic compounds (VOCs) on the sensory characteristics of beverages is essential for enhancing the overall sensory quality of alcoholic fruit beverages (AFBs). The perception of these sensory attributes arises from the interaction between volatile compounds and human sensory receptors. Furthermore, the VOCs found in a ‘high-quality’ alcoholic beverage tend to be diverse and nuanced. However, only a subset of these compounds—specifically those present at levels exceeding their sensory detection thresholds—meaningfully contribute to the aroma profile of AFBs [75]. Interactions between volatile and non-volatile components, including synergistic and antagonistic effects, also influence mouthfeel and taste perception. Therefore, identifying the key VOCs in AFBs, particularly those directly linked to perceivable sensory attributes, is a central objective in fruit-based beverage research. Yeast strains constitute a critical factor in shaping the complexity and sensory attributes of fermented beverages. It is well established that these microorganisms are responsible for the biosynthesis of a wide array of secondary metabolites, which play a decisive role in determining the final aroma and flavor profile of the product [76]. Volatile compounds can be classified according to their chemical class into higher alcohols, esters, aldehydes, ketones, volatile acids, and other volatiles (terpenes and sulfur compounds) [77,78]. Moreover, VOCs can be classified according to their origin as primary, secondary, and tertiary compounds. Primary VOCs originate directly from fruit tissues and constitute the varietal aroma of the raw material. Their distribution may differ among fruit tissues: the pericarp (skin or peel) is typically enriched in terpenoids, norisoprenoids, and aldehydes that contribute floral, citrus, herbaceous, and resinous notes, whereas the flesh generally contains higher proportions of esters, alcohols, aldehydes, and lactones associated with fruity and sweet aromas generated during fruit ripening [79,80]. Secondary VOCs are formed during alcoholic fermentation through microbial metabolism, particularly by yeasts, and include esters, higher alcohols, volatile fatty acids, carbonyl compounds, sulfur-containing compounds, and other fermentation-derived aroma molecules that greatly influence beverage sensory quality [1,9,79,81]. Tertiary VOCs arise during post-fermentation processes such as maturation and aging through chemical and biochemical transformations involving both fruit- and fermentation-derived compounds [79]. Ester molecules are compounds formed through the reaction between the hydroxyl group of an alcohol and the carboxyl group of organic acids. These substances play a direct role in shaping the organoleptic characteristics and sensory perception of wines, particularly contributing to fruity notes [79,82]. The overall concentration of esters in wine is typically substantial and often exceeds their sensory threshold, thereby significantly affecting the final sensory profile [81]. Although more than 150 types of esters can be identified in wine, most occur at extremely low levels and have little impact on their overall aroma. Numerous factors affect the formation of esters, including the characteristics of the fruit, fermentation conditions and the surrounding environment [79]. Furthermore, the microbial community plays a central role in ester formation, given that different yeast species have varying enzymatic capacities for ester biosynthesis. *S. cerevisiae* is characterized by its propensity to produce elevated levels of esters. Non-*Saccharomyces* yeasts, including *W. saturnus*, have been observed to exhibit notably elevated levels of acetate ester production. It has been demonstrated that co-fermentation and sequential inoculation strategies result in a substantial enhancement of acetyl and ethyl ester production. This, in turn, has been shown to increase the overall bouquet intensity and complexity of the beverage [44]. Studies conducted on fruit-based fermentations consistently show differences in volatile composition between monoculture and mixed-culture fermentations. Single-strain fermentations, particularly those performed with *S. cerevisiae*, generally produce volatile profiles dominated by ethyl esters, acetate esters, and higher alcohols, resulting in predictable but less complex aroma profiles [19,26,79]. In contrast, mixed fermentations involving *Saccharomyces* and non-*Saccharomyces* species frequently increase both the concentration and diversity of aroma compounds. For example, co-fermentation of papaya juice with *S. cerevisiae* and *W. saturnus* increased the abundance of esters, alcohols, aldehydes, and fatty-acid-derived volatiles compared with single-strain fermentations [63]. Similarly, mixed fermentations of mango, apple, pear, fig, cherry, and durian matrices have been associated with higher concentrations of acetate esters, ethyl esters, higher alcohols, terpenes, and other aroma-active compounds, resulting in greater sensory complexity and improved fruity and floral characteristics [33,34,44,47,64,65,66,67,68]. Differences are also observed between fermentations driven exclusively by yeasts and those involving non-yeast microorganisms. While yeasts are the principal producers of ethanol, esters, higher alcohols, volatile thiols, and many terpene-derived aroma compounds, lactic acid bacteria and acetic acid bacteria primarily contribute through the production or transformation of organic acids, carbonyl compounds, and other metabolites that modify aroma perception, acidity, and mouthfeel [51,83]. In mixed microbial fermentations, these organisms may interact synergistically with yeasts, leading to greater flavor complexity than that achieved by yeast-only fermentations [51,83]. Higher alcohol contributes significantly to the organoleptic complexity. *T. delbrueckii* is distinguished by its ability to produce higher alcohols [44]. The increased production of pleasant-smelling 2-phenylethyl alcohol is a characteristic contribution of species such as *M. pulcherrima*, *L. thermotolerans*, and *S. bacillaris* [9].

It should be noted that the current understanding of yeast-mediated formation of volatile thiols and terpene-derived aroma compounds is derived predominantly from grape wine research [84,85]. While these mechanisms provide a valuable framework for interpreting aroma development in fermented fruit beverages, direct evidence in non-grape fruit matrices remain comparatively limited [79]. In contrast, studies on fermented apple, pear, cherry, fig, mango, papaya, lychee, and durian beverages have mainly focused on global changes in volatile composition and sensory properties following the use of selected *Saccharomyces* and non-*Saccharomyces* starters, rather than on the specific metabolism of thiol and terpene precursors [86]. Therefore, caution is required when extrapolating mechanistic conclusions from wine to other fruit-based fermentations.

Volatile thiols, such as 4-mercapto-4-methylpentan-2-one (4MMP), 3-mercaptohexan-1-ol (3MH), and 3-mercaptohexyl acetate (3MHA), contribute tropical, passion fruit, and grapefruit aromas. Volatile thiols, which are largely absent from grape juice, are produced during fermentation from odorless precursors by carbon-sulfur lyases produced by certain non-Saccharomyces yeasts, including *P. kluyveri* and *S. bacillaris* [9] and then from non-volatile precursors present in the must [84]. Several studies have shown that 4MMP and 3MH occur in grapes in the form of non-volatile precursors, which are bound either to cysteine or glutathione. The main enzyme responsible for cleaving cysteine-conjugated precursors is *S. cerevisiae* β-lyase IRC7, which exhibits a stronger substrate preference for cys-4MMP than for cys-3MH [87]. The pathway by which glutathione-linked thiol precursors are metabolized involves several enzymatic steps and is thought to proceed through the formation of cysteinylated intermediates [85]. Both 4MMP and 3MH occur in grapes as non-volatile cysteinylated and glutathionylated precursors, which are enzymatically cleaved during fermentation to release the corresponding volatile thiols [84,85,87]. Subsequently, 3MH can be converted into 3-mercaptohexyl acetate (3MHA), a compound characterized by intense tropical fruit aromas, through acetylation catalyzed by alcohol acetyltransferases such as ATF1 [88].

Monoterpenoids (C10 compounds), sesquiterpenoids, and C13 norisoprenoids, which belong to the isoprenoid class, are important for the aroma of wine and fermented beverages. The role of yeasts, especially *S. cerevisiae*, in releasing monoterpenoids through enzymatic action during fermentation has been a subject of discussion for many years. Although early studies demonstrated that *S. cerevisiae* exhibits measurable β-glucosidase activity [89], this activity is substantially lower than that reported for numerous non-*Saccharomyces* yeast species [90]. Moreover, grape-derived β-glucosidases function optimally at around pH 5 and are heavily inhibited by glucose and ethanol [91]. This suggests that they contribute minimally to the liberation of monoterpenoids from glycosidic precursors during fermentation.

Various extracellular hydrolytic enzymes, including β-glucosidase, α-arabinosidase, α-rhamnosidase, α-xylosidase and α-apiosidase, have been identified in *S. cerevisiae* and non-*Saccharomyces* yeasts [92]. It has also been suggested that exo-β-glucanases may be involved in the cleavage of monoterpenoid glycosides [80]. For instance, in Gewürztraminer wine, the combination of *T. delbrueckii* and *S. cerevisiae* led to higher levels of α-terpineol and linalool, which are important aroma compounds. However, wines fermented only with *S. cerevisiae* had higher amounts of nerol and geraniol. Overall, the mixed fermentation improved the wine’s quality.

Strains belonging to *M. pulcherrima* species are also known to produce β-D-glucosidase, which can increase levels of α-terpineol, nerol, and geraniol when used alone. However, when it is used together with *S. cerevisiae*, the levels of nerol and geraniol are lower than expected, and only α-terpineol increases [93]. This happens because *S. cerevisiae* can convert nerol and geraniol into α-terpineol during fermentation. This shows how interactions between different yeasts can strongly affect the final aroma [9].

Regarding *Debaryomyces* species, a strain of *Debaryomyces vanriji* isolated from grapes was shown to influence the aroma of Muscat of Frontignan wine when used together with *S. cerevisiae* [94]. Wines produced with this mixed culture had different levels of aroma compounds, including terpenes, compared to control wines. In particular, the increase in geraniol was linked to the activity of β-D-glucosidase produced by *D. vanriji*, which helps release aroma compounds from their bound forms. These wines also showed higher enzyme activity during fermentation and lower levels of bound geraniol.

Beyond the enhancement of desirable compounds, the objective of yeast selection is also to reduce the production of undesirable compounds. High concentrations of acetaldehyde (>100 mg/L) have been shown to have a detrimental effect on wine flavor, with the result that certain yeast strains are excluded from use [4]. It has been observed that certain non-*Saccharomyces* species (e.g., *Hanseniaspora*, *Zygosaccharomyces*) have the capacity to produce elevated levels of acetic acid, although low-producing strains within these genera are also present and are actively sought after [9]. In addition, *S. cerevisiae* strains can be screened and selected in order to reduce the production of hydrogen sulfide (H_2_S), which is associated with the production of unpleasant rotten-egg or sewer smells [12].

Overall, the available evidence indicates that yeast selection has a major influence on aroma development in fermented fruit beverages. However, the strength of evidence differs among compound classes. Direct experimental data from non-grape juice fermentations clearly demonstrate yeast-dependent modulation of esters, higher alcohols, organic acids, and overall sensory complexity in products such as cider, pear, cherry, fig, mango, papaya, lychee, and durian fermented beverages. By contrast, much of the mechanistic understanding regarding volatile thiol release and terpene biotransformation derives from grape wine studies, where precursor molecules and enzymatic pathways have been extensively characterized. Consequently, while strains belonging to *P. kluyveri*, *T. delbrueckii*, and *M. pulcherrima* species are recognized as promising tools for enhancing aroma complexity across fruit fermentations, further studies are needed to confirm the occurrence, precursor availability, and metabolic routes of volatile thiols and terpenes in non-grape fruit matrices [26,33,34,44,47,63].

### 5.3. Influence on Acidity, Mouthfeel, and Color Stability

Beyond the parameters of aroma and ethanol content, the selection of yeast has been demonstrated to exert a profound influence on other critical quality parameters of fermented fruit beverages, including acidity, mouthfeel, and color stability. Yeasts have been shown to play a significant role in modulating the acidity of the final product. Non-*Saccharomyces* yeasts, for instance, have been shown to reduce volatile acidity (e.g., *T. delbrueckii* and *M. pulcherrima* are known to reduce acetic acid levels). In contrast, certain strains, such as *L. thermotolerans*, have been observed to function as acidifying agents through the production of L-lactic acid. It has been demonstrated that other organisms, for instance *Sc. pombe*, are capable of deacidification through the process of consuming malic acid. This enables precise regulation of the beverage’s overall acid balance [9].

The perception of mouthfeel is significantly influenced by the concentration of glycerol. It is a commonly held view that an elevated level of glycerol in wine is indicative of an enhancement in the overall taste balance and a fuller mouthfeel. It is evident that certain non-Saccharomyces yeasts, most notably *M. pulcherrima*, are distinguished by their capacity to yield wines that exhibit elevated levels of glycerol [53]. In fact, glycerol concentrations of 10.1 g/L and 11.5 g/L were reported in sequential fermentations with *M. pulcherrima* strains AWRI1149 and AWRI1656, respectively, compared with 6.2 g/L in the *S. cerevisiae* control strain. Furthermore, in grape must fermentations, sequential inoculation with the AWRI1149 yeast strain yielded 8.1 g/L glycerol in Chardonnay and 14.9 g/L in Shiraz wines, confirming the strain’s ability to substantially enhance glycerol production. The ability of yeast strains to contribute to the color stability of the final product has been well documented [9]. The effect of co-fermentation on color is subject to variation, as demonstrated in several studies which have indicated an increase in its intensity in specific co-fermented wines [57]. However, it is also known that yeast cell walls have the capacity to adsorb phenolic compounds and other pigments, which may result in color loss. It is therefore imperative that strains exhibiting low pigment adsorption are subject to rigorous screening to ensure the maintenance of desired color stability. Furthermore, various yeast enzyme activities, such as β-glucosidase, anthocyanidase, and pectinolytic enzymes, along with the production of polysaccharides like mannoproteins, also influence wine color and its long-term stability [4].

While the importance of aroma in the development of beverages is frequently emphasized, the extant literature indicates that the selection of yeast has a profound effect on other critical quality parameters, including acidity, mouthfeel and color stability. These elements, it should be noted, represent more than mere supplementary considerations; rather, they constitute indispensable components of a beverage of superior quality, thereby significantly contributing to the overall consumer experience [4]. The demonstrated ability of yeasts to modulate these attributes, for example, by producing glycerol for improved mouth-feel or by secreting enzymes for enhanced color extraction and stability, highlights their overall influence on the final product. Although higher glycerol concentrations may contribute to increased viscosity, body, and perceived sweetness, mouthfeel is a multifactorial attribute that is also influenced by yeast-derived polysaccharides (e.g., mannoproteins), ethanol, organic acids, and aroma-active compounds [95]. Consequently, improvements in palate fullness cannot be attributed to glycerol alone. In addition, yeasts can influence color stability through anthocyanin adsorption onto cell walls and through the production of metabolites such as pyruvic acid and acetaldehyde, which promote the formation of stable pyranoanthocyanins [96]. These effects are highly strain dependent and contribute to differences in both sensory and chromatic characteristics of fermented beverages.

### 5.4. Nutritional and Functional Enhancements

In addition to their role in the production of alcoholic beverages, fermentation processes, particularly those involving selected yeast strains, offer significant opportunities for enhancing the nutritional and functional properties of fruit-based products.

Fermentation has been shown to enhance the nutritional value of fruit wines by facilitating the release of amino acids and other essential nutrients from the yeast cells themselves [2]. Fermentation is recognized as an effective method of preventing fruit spoilage. The process described herein results in the formation of newly derived compounds through the interaction between microbes and fruit nutrients. This interaction has been shown to enhance color, increase the content of active ingredients, and improve the overall nutritional value of the fermented fruit [51].

Fermentation can thus be leveraged to produce functional beverages and foods that are enriched with probiotics, antioxidants [97], and other bioactive compounds. The specific functional benefits are contingent on the fruit matrix being fermented and the types of microorganisms employed [6]. For instance, *S. cerevisiae* has been shown to accumulate trace elements such as selenium, zinc, and chromium, which offer potential health benefits when incorporated into fermented products [4]. Examples from typical Thai fermented foods, such as “Khao mak” (fermented rice), illustrate these benefits; it contains probiotics, organic acids, vitamins, and minerals, which are associated with improved digestion, enhanced antioxidant capacity, and the alleviation of various ailments [38].

The emphasis on “functional activities” [51] and “health benefits” [4] derived from fermented fruits represents a significant trend in the food industry. This encompasses the incorporation of probiotics into products, the augmentation of antioxidants [97], vitamins, and other bioactive compounds. This development signifies a notable shift towards utilizing fermentation as a biotechnological instrument to produce nutraceutical products, as opposed to its application in the production of alcoholic beverages. The emergence of this novel role has the potential to broaden the market scope for fermented fruit beverages, extending beyond conventional consumption patterns. These beverages can be positioned as health-promoting functional foods, a concept that is a significant catalyst for innovation and product diversification within the contemporary food industry.

## 6. Recent Advances and Future Perspectives

### 6.1. Novel Yeast Strains and Their Applications

The continuous search for new yeast strains with optimal technological applications remains a central focus in the industry [1]. A notable trend is the increasing preference for indigenous yeast strains. These strains have adapted naturally to the specific micro-environmental conditions of their geographical origin and possess the unique ability to preserve distinctive sensory attributes, thereby contributing to the “terroir” of a product [4].

Increasing evidence suggests that indigenous microbial communities may contribute to the concept of ‘microbial terroir’. Several studies have shown that grape-associated yeast populations differ according to geographical origin and vineyard location, and that these region-specific microbial communities can influence fermentation behavior and wine aroma composition. Consequently, the use of indigenous strains may contribute to the expression of regional sensory characteristics and product typicity [98,99,100,101].

For instance, indigenous *S. cerevisiae* strains isolated from Guichang kiwi have demonstrated superior sensory attributes and aroma quality in comparison to commercial strains. These strains have been shown to produce wines with lower total organic acids and more pronounced floral and fruity aromas [22]. In a similar manner, in the context of cider production, indigenous strains have been identified as optimal for enriching local products with enhanced quality [13].

In addition to the use of indigenous yeast strains, novel applications are being developed for existing species. An interesting development is the use of Sake yeast strains of *S. cerevisiae* for cider production, which has led to beverages with excellent organoleptic properties and enhanced fruitiness [102]. This demonstrates a cross-application of specialized strains to new fruit matrices. The use of sake yeast strains in cider production has attracted attention because these strains exhibit metabolic characteristics that differ from those of conventional yeast strain for cider production. In particular, sake yeasts are often associated with elevated production of aroma-active esters, including ethyl caproate and isoamyl acetate, which impart apple-, banana-, and tropical fruit-like aromas [103].

Comparative studies have shown that ciders fermented with sake yeasts may exhibit enhanced fruity and floral sensory attributes, greater intensity, and a more complex volatile profile than those produced with conventional cider strains. These properties make sake yeasts promising alternatives for the development of differentiated cider styles and novel sensory profiles [104].

Beyond this, research efforts are expanding to isolate and characterize yeasts from new and underutilized fruits. Studies on Amazonian native fruits (e.g., aguaje, camu camu) and various coffee fruits aim to identify novel yeast species and strains with unique biotechnological potential for fermentation [35].

The innovation in yeast utilization is characterized by a dual focus: a “local-global nexus.” On one hand, there is a clear trend towards exploring and utilizing “indigenous” or “native” yeast strains from specific fruits or geographical regions [4]. These strains are often better adapted to their native environments and can impart unique “terroir” characteristics, which are crucial for product differentiation and establishing regional identity. On the other hand, there is a growing interest in applying yeasts from one specific fermentation domain, such as Sake yeasts, to entirely new fruit matrices, like cider [102]. This simultaneous exploration of local biodiversity and innovative cross-domain application of known strains represents a dynamic and highly innovative approach to product development. This trend suggests a future where fermented fruit beverages are increasingly diversified, offering both unique regional expressions using native strains and novel product categories through the creative and innovative cross-applications of well-characterized strains.

### 6.2. Genomic and ‘Omics’ Approaches

Advancements in microbiology and biotechnology have had a considerable impact on the field of fermentation, particularly through the development of sophisticated starter cultures [6]. The integration of high-throughput ‘omics’ technologies has been instrumental in this regard. Phylogenomics, defined as the comparative analysis of hundreds of non-*Saccharomyces* yeast genomes, provides unprecedented insights into their metabolic networks. This comprehensive genomic knowledge enables researchers to comprehend the way these yeasts contribute to fermentation capacity and regulate flavor and aroma profiles [31].

A pioneering aspect of this research is the identification of the ‘fermentome’ and ‘flavorome’. The non-*Saccharomyces* ‘fermentome’ is defined as a specific set of genes (e.g., six genes for the strict fermentome, 35 for the complete one) that are uniquely present in fermentative species and absent in non-fermentative ones, thus providing a genetic basis for their fermentative capabilities. A ‘flavorome’ has also been delineated, comprising 96 genes across 19 metabolic categories that are directly implicated in wine aroma and flavor enhancement. These findings offer invaluable insights into the genome, enabling more precise strain selection and fostering innovative winemaking practices [31]. The identification of the non-Saccharomyces fermentome and flavorome provides a genomic framework for linking specific genes to fermentation performance and aroma production. The fermentome includes genes associated with sugar utilization, ethanol production, and stress adaptation, while the flavorome encompasses genes and enzymatic functions involved in the synthesis or release of aroma-active compounds such as esters, terpenes, and higher alcohols. Consequently, these genetic signatures can be used as molecular markers to predict oenologically relevant phenotypes and guide the selection of strains with desirable technological and sensory traits before extensive fermentation trials [55,105]. In addition, integrated multi-omics approaches combining genomics, transcriptomics, proteomics, and metabolomics enable a deeper understanding of the molecular determinants underlying yeast performance and flavor formation, facilitating the development of tailored starter cultures for specific winemaking objectives [106]. The technologies under discussion facilitate the identification of specific genes and enzymes associated with flavor and aroma production. Furthermore, they enable the detection of elevated levels of bioactive compounds in fermented products, thereby providing a comprehensive overview of the metabolic contributions of yeast [38].

The advent of concepts such as the “fermentome” and “flavorome”, derived from large-scale phylogenomic analyses, represents a substantial progression from empirical observation to data-driven approaches in yeast selection. Rather than relying exclusively on trial-and-error screening, researchers can identify candidate genes and metabolic pathways potentially associated with desirable technological traits. However, the expression of fermentation-related phenotypes depends on complex interactions among genetic background, regulatory networks, and environmental conditions, and therefore cannot always be accurately inferred from genomic information alone [107]. Consequently, omics approaches should be viewed as powerful tools for generating hypotheses and guiding strain selection, but their predictions require validation through phenotypic and fermentation studies conducted under application-relevant conditions [108].

Future developments in yeast selection are expected to move beyond the individual application of genomics, transcriptomics, proteomics, and metabolomics toward integrated multi-omics and systems biology approaches. The combination of these datasets will facilitate a more complete understanding of the complex interactions linking genotype, gene expression, metabolism, and fermentation phenotypes [7,38].

In parallel, advances in genome-scale metabolic modeling and machine learning are expected to improve the identification of candidate strains and the prediction of desirable technological traits [109]. Precision fermentation strategies, supported by synthetic biology, metabolic engineering, and advanced breeding techniques, may further enable the development of starter cultures with tailored characteristics, including optimized aroma production, reduced ethanol yield, enhanced stress tolerance, and improved utilization of alternative fruit substrates [74].

These approaches may also contribute to the valorization of underutilized fruits and agricultural by-products within sustainable production systems. Nevertheless, the successful implementation of these technologies will require careful validation under industrially relevant conditions and consideration of economic, regulatory, and consumer-acceptance aspects [7].

### 6.3. Biocontrol Applications and Reduction in Undesirable Compounds

A significant recent advance is the increasing recognition of non-*Saccharomyces* yeasts for their biocontrol capabilities. These yeasts have been shown to be effective in combating fungal pathogens in vineyards and in restraining the proliferation of spoilage yeasts during the winemaking process. This suggests that they could offer a sustainable alternative to chemical interventions. Non-*Saccharomyces* yeasts have been shown to produce a variety of antimicrobial metabolites, with killer toxins (KTs) being the most notable. These KTs have been shown to inhibit susceptible yeast strains and often exhibit a broader inhibition spectrum and greater stability compared to KTs produced by *S. cerevisiae* [23].

Illustrative examples comprise KTs from *W. anomalus* and *T. delbrueckii*, which have exhibited efficacy against spoilage yeasts such as *Brettanomyce*s [23]. The use of selected protective cultures from the same ecological niche as wine fermentation can prove to be particularly advantageous, a phenomenon attributable to their natural adaptation and competitive strategies. This approach constitutes a promising and cost-effective alternative for reducing reliance on sulfites (SO_2_), which are a growing concern due to their associated health implications and the increasing tolerance of spoilage yeasts to these compounds. In addition, non-Saccharomyces yeasts are valuable for their enzymatic activity.

It has been established that a wide variety of extracellular depolymerizing enzymes are produced, including pectinases, cellulases, xylanases and glycosidases. These enzymes have been demonstrated to enhance wine sensory profiles and improve technological processes, thereby reducing the necessity for costly exogenous enzyme additives [23].

The conventional approach to spoilage control in fermentation has historically relied heavily on the use of chemical additives such as sulfur dioxide (SO_2_) [1]. Nevertheless, mounting concerns regarding potential health implications, coupled with the escalating microbial tolerance to these chemicals, are prompting an intensive search for more natural alternatives. The identification and subsequent employment of non-*Saccharomyces* yeasts as biocontrol agents, through their capacity to produce killer toxins or various enzymes [23], signifies a substantial transition towards a more biological and sustainable approach. The utilization of the natural microbiological ecology is essential in ensuring the maintenance of product quality and safety. This trend has a substantial impact on the development of “clean label” products and the promotion of sustainable production practices. These practices are in alignment with the growing consumer demand for natural ingredients and environmentally friendly processes.

### 6.4. Sustainability and Revalorization of Fruit By-Products

Fermentation is proving to be a key strategy for enhancing sustainability within the food supply chain, particularly through the revalorization of horticultural by-products. This encompasses materials such as fruit peels, seeds, pomace, and even non-aesthetic or unmarketable fruits that would otherwise be discarded [6]. Fermentation is a process which has the capacity to minimize food loss and waste, thereby ensuring that the value derived from agricultural resources is maximized.

This process has been shown to address issues of waste reduction, while concomitantly enhancing the nutritional value, digestibility, safety, and sensory properties of food products derived from these by-products. The process of fermentation facilitates the production of innovative food products and functional beverages, derived from fruit materials that were previously regarded as discarded or unmarketable.

Notwithstanding their considerable potential, most studies on non-Saccharomyces yeasts and mixed fermentations remain confined to laboratory-scale systems. Scale-up can be challenging because oxygen transfer, nutrient availability, and microbial interactions may differ substantially under industrial conditions, affecting strain competitiveness, fermentation kinetics, and aroma production. Furthermore, maintaining consistent microbial population dynamics and reproducible product quality at large scale remains difficult, making pilot-scale validation a crucial step prior to commercial application [110]. Moreover, the seasonality of by-products and their dependence on specific crops and processing times frequently constrain the scope of these studies [6]. Future research in this area should prioritize the development of scalable fermentation models to facilitate their industrial implementation. Furthermore, there is a significant need to explore the potential of under-utilized by-products to maximize the overall impact of fermentation, especially in regions facing challenges related to food accessibility. The emphasis on the “revalorization of horticultural by-products” [6] through the process of fermentation directly addresses critical issues of food waste and resource utilization. This development signifies a discernible transition towards a circular economy model, wherein waste streams are not merely disposed of, but rather undergo active transformation into valuable products [111]. This process has been shown to engender substantial environmental benefits through the reduction in waste, while concomitantly generating new revenue streams and enhancing overall sustainability throughout the entire food supply chain. Consequently, fermentation is positioned as a critical technology for developing sustainable food systems. This is because it contributes simultaneously to both environmental stewardship and new economic opportunities derived from low-value input.

### 6.5. Recent Starter Applications for Fermented Beverages Production from Fruit Juices

A thorough review by Pinto et al. [112] provides an in-depth discussion of consumer acceptance, chemical and sensory characteristics of fruit juices and fruit fermented beverages. While the text does provide a comprehensive overview of fruit fermentation, it also offers a contextual analysis of the growing interest in fruit fermentations. In doing so, it emphasises their potential health benefits and the use of non-thermal processing methods to preserve nutritional and bioactive compounds.

In the recent study, Yuan and colleagues [83] provide a comprehensive overview of recent advances in the field of fermented fruits, with a particular emphasis on the analysis of strains, the examination of fermentation strategies, and the assessment of functional activities. The text goes on to explore the beneficial effects of fruit fermentation, including the extension of shelf life, the enhancement of flavour and colour, and the increase in active ingredients. The review under consideration here draws attention to the use of various micro-organisms, including lactic acid bacteria, acetic acid bacteria, and yeasts (both *Saccharomyces* and non-*Saccharomyces* species), in the fermentation of different fruit types, including black wolfberry, blueberry, pumpkin, and tomato. The text under consideration places particular emphasis on the way, microbial fermentation has been demonstrated to enhance the nutritional value of a given substance by increasing polyphenols, flavonoids, organic acids and vitamins. It also explores how specific strains of micro-organisms possess the capacity to impart distinctive aromas to the substance, which in this instance are described as fruity and floral.

A study by Ferreira et al. [113] provides a bibliometric analysis, technological review and market perspectives on low-alcohol fruit wines. This work emphasises the mounting utilisation of non-conventional fruits for value addition, particularly due to their distinctive chemical compositions and potential health benefits. The study emphasises the decisive role of factors such as fruit species and maturation stage in influencing ethanol production and the overall quality of the beverage.

In their subsequent investigation, Keșa et al. [21] explore the functional aspects in greater depth, examining strategies to enhance the potential functionality of fruit-based fermented beverages. This review underscores the functional potential of both alcoholic and non-alcoholic fermented fruit beverages, considering their natural occurrence and enrichment in phenolic compounds, vitamins, and minerals. In addition, an evaluation of pre-fermentative methodologies employed for the optimisation of bioactive compound extraction from fruits is conducted, encompassing maceration techniques and an array of extraction methodologies. As illustrated by the following specific examples, there is a broad range of fruit applications and associated health benefits such as fermented jabuticaba berry beverage, pomegranate fermented juice, sea buckthorn wine and symbiotic fermented Cornelian cherry beverage.

In a recent publication, Suhre et al. [114] explored the fermentation of native Brazilian fruits with kombucha, presenting it as a novel opportunity for producing low-alcohol beverages. The present research focuses on the utilisation of multi-species starter cultures from kombucha, composed of yeasts and acetic acid bacteria, for the development of new functional beverages from diverse fruit substrates. The study posits that kombucha cultures exhibit a high degree of adaptability in their capacity to ferment a variety of fruits, thereby suggesting the potential for health benefits that encompass antimicrobial, antioxidant, anticancer, and antidiabetic properties.

Regarding fruit categories, research on cider (i.e., fermented apple juice) is a constantly evolving field. A study highlighted by Zhou et al. [115] investigates the effect of mixed-culture fermentation (using *S. cerevisiae*, and *P. kudriavzevii*) yeasts during cider fermentation. The findings of the study indicate that triple mixed-culture fermentations result in a reduction in acid and alcohol content, an enhancement of antioxidant activities, and an improvement of flavour and aroma profiles with higher ester compounds. This ultimately leads to superior sensory qualities in comparison to single-culture fermentations.

Concurrently, research on perry, a fermented pear juice, is also undergoing significant advancement. Liu et al. [116] investigated the impact of glycosidases and GSH pretreatments, fermentation temperatures, and ageing time on the physicochemical, organic acid, and aroma profiles of perry. This study underlines the potential to enhance the quality of perry through the optimisation of fermentation conditions and pretreatments. This contributes to a fuller understanding of the factors that influence the final characteristics of the beverage. A further study by de Garcia et al. [117] concentrated on spoilage yeasts in perry production in Argentina. This study made a significant contribution to the development of control strategies to prevent microbiological contamination and minimise product loss in this emerging market. Another promising technological approach for improving the efficiency of fruit beverage production is the use of immobilized yeast cells. Immobilization consists of the physical confinement of viable microbial cells within or onto suitable carrier materials while maintaining their metabolic activity. Immobilized yeast systems have attracted increasing interest because they allow cell reuse, facilitate biomass recovery, improve process control, and may shorten fermentation times. In addition, immobilized cultures often exhibit enhanced tolerance to environmental stresses such as high ethanol concentrations, low pH, and temperature fluctuations. These characteristics can contribute to improved fermentation reliability and greater product consistency at industrial scale. Immobilized *S. cerevisiae* and non-*Saccharomyces* starter cultures have been investigated to produce wines, ciders, fruit wines, and other fermented beverages, showing potential for continuous and repeated-batch fermentation processes. Despite these advantages, further research is required to optimize carrier materials, evaluate long-term cell stability, and assess economic feasibility under commercial production conditions [118,119,120]. Taken together, these studies demonstrate a growing trend in the utilisation of a wide array of fruits, beyond grapes, for the purpose of fermentation, driven by consumer demand for healthier, novel, and functional beverages. Researchers are actively exploring diverse microbial strains, innovative fermentation techniques, and pre-fermentative treatments to optimise the sensory, nutritional, and health-promoting properties of these fermented fruit juices, and current evidence indicates that fruit fermentation is evolving beyond historically established preservation processes toward the development of functional and value-added beverages. Recent reviews have highlighted the growing use of diverse fruit matrices and microbial consortia to improve product quality, extend shelf life, and enhance the concentration and bioavailability of health-promoting compounds, including polyphenols, flavonoids, vitamins, and organic acids [21,83,112].technological innovations such as mixed-culture fermentations, kombucha-based fermentations, and optimized pre-fermentative treatments have been shown to enhance aroma complexity, antioxidant activity, and sensory acceptance Research on specific fermented products, including cider and perry, further emphasizes the importance of microbial selection, fermentation conditions, and spoilage control in shaping the chemical and sensory characteristics of the final beverage [115,116,117]. Overall, current evidence indicates that the integration of novel fruit substrates, tailored microbial cultures, and process optimization strategies offers significant opportunities for producing innovative fermented beverages that meet increasing consumer demand for sustainable, functional, and sensory appealing products.

## 7. Conclusions

The field of fermented fruit beverages is undergoing a profound transformation, propelled by sophisticated advancements in yeast technology and biotechnology. This report emphasized the crucial and complex functions of both *Saccharomyces* and non-*Saccharomyces* yeasts in determining the sensory, chemical and functional characteristics of these products.

*S. cerevisiae* is an indispensable tool due to its capacity for ethanol production and predictable fermentation. However, contemporary research has transformed it also into a designed instrument, with efforts focused on engineering strains for specific outcomes, including targeted aroma profiles, reduced ethanol content, and enhanced stress tolerance. Concurrently, non-*Saccharomyces* yeasts have transitioned from being perceived as mere contaminants to being recognized as invaluable contributors to flavor complexity, analytical composition, and even biocontrol, often employed in synergistic mixed-culture fermentations.

The methodologies employed for yeast selection have also evolved, progressing from basic phenotypic screening to advanced molecular and ‘omics’ approaches. The capacity to discern a yeast’s ‘fermentome’ and ‘flavorome’ furnishes unparalleled genomic insights, thereby facilitating a more precise and predictive approach to strain selection and engineering. This transition from a conventional empirical trial-and-error approach to a data-driven design methodology has been shown to facilitate accelerated product development while ensuring enhanced consistency.

The strategic utilization of yeast in co-fermentation and sequential inoculation strategies has shown considerable potential for improving fermentation performance and sensory quality in several fruit-based beverages. This allows for the creation of complex and unique flavor profiles that are unattainable with single strains. These approaches are based on the utilization of synergistic interactions and temporal control over yeast metabolism, with the objective of achieving precise modulation of the characteristics of the resulting beverages. These solutions achieve this by enabling the reuse of cells and reducing the amount of energy required.

In addition to sensory considerations, the selection of yeast has become increasingly significant in the development of functional beverages that are enriched with antioxidants, and other bioactive compounds. This has the effect of expanding the market potential for fermented fruit products as health-promoting foods. Furthermore, the process of consequently resulting in a more extensive range of high-quality, diversified, and sustainably produced fermented fruit beverages that will cater to the evolving consumer demands fermentation is of major significance in the context of the circular economy, as it results in the revalorization of horticultural by-products and contributes to a substantial reduction in waste, thereby promoting sustainable resource management. In conclusion, the field of fermented fruit beverages is characterized by dynamism and rapid innovation.

The ongoing exploration of yeast biodiversity, in conjunction with state-of-the-art genomic and biotechnological tools, signifies the potential for additional breakthroughs, consequently resulting in a more extensive range of high-quality, diversified, and sustainably produced fermented fruit beverages that will cater to the evolving consumer demands. Despite the significant advances achieved through genomics, omics technologies, and innovative screening approaches, the industrial implementation of novel starter cultures still presents important challenges. The routine application of high-throughput omics tools may be constrained by costs, infrastructure requirements, and the need for specialized bioinformatic expertise, which can limit their adoption outside large research centers and industrial laboratories [7]. Moreover, strains showing promising results under laboratory conditions are not always commercially available or suitable for large-scale production [108]. Industrial starter cultures must also demonstrate genetic and phenotypic stability during propagation and storage, as well as robustness under variable processing conditions typical of commercial fermentations [18]. Moreover, inadequate adaptation of selected strains to industrial environments may increase the risk of sluggish or stuck fermentations, which remain among the most important technological challenges affecting fermentation efficiency, product quality, and economic sustainability [121]. Consequently, successful industrial adoption requires not only desirable metabolic and sensory traits but also process reliability, scalability, compatibility with existing production practices, and economic feasibility [6]. Future research should therefore focus on bridging the gap between laboratory-scale characterization and commercial application through validation under industrially relevant conditions.

## Figures and Tables

**Table 1:** Key Characteristics of Selected *Saccharomyces* and Non-*Saccharomyces* Yeast Species.

Yeast Species	Primary Fermentative Role (in Grape Must)	Key Aroma/ Flavor Contributions	Stress Tolerance	Other Notable Contributions	Reference
*Saccharomyces cerevisiae*	High ethanol production (11–13% *v*/*v*)	Higher alcohols, fatty acids, esters (fruity, floral notes); 2-phenylethanol, isoamyl acetate	Ethanol > 12%, osmotic, pH > 4, temperature fluctuations	Biomass generation; reduced ethanol (with glycerol); mycotoxin adsorption; killer toxin production [4]	[1,4]
*Torulaspora delbrueckii*	Complete alcoholic fermentation, lower glycerol/acetic acid	Higher alcohols, isoamyl acetate, terpenes; ethyl caprylate; 2-phenylethyl alcohol	Ethanol < 12%, pH > 4, osmotic	Low volatile acidity; biocontrol (killer toxins against *Brettanomyces*); pectinase activity; increased color extraction [8]	[8,9,10]
*Metschnikowia pulcherrima*	Partial fermentation, high glycerol	Pleasant 2-phenylethyl alcohol; influences higher alcohols; color stability	Ethanol < 12%, pH > 4, osmotic [8]	Biocontrol (iron depletion); pectinase, esterase, protease activity; reduced acetic acid	[8]
*Hanseniaspora uvarum*	Dominant in early stages, some acetic acid	β-D-glucosidase for terpenes; 2-phenylethyl acetate, isoamyl acetate	Ethanol < 12%, pH > 4, osmotic [8]	Biocontrol potential; high catalase activity	[8,9]
*Lachancea thermotolerans*	L-lactic acid production; 8–9% ethanol	Increased lactic/succinic acids; 2-phenylethyl alcohol; better antioxidant activity	Ethanol > 12%, pH > 4, osmotic	Low volatile acidity; biocontrol potential [8]	[8,9,10]
*Pichia kluyveri*	Can produce high 3-mercaptohexan-1-ol and 3-mercaptohexyl acetate; some ethyl acetate	Volatile thiols (tropical aromas); 2-phenylethyl acetate, isoamyl acetates	Ethanol > 12%, pH > 4, osmotic	β-Glucosidase activity; protease activity	[8,9]
*Williopsis saturnus*	Produces acetate esters	High ethyl esters (hexanoate, decanoate, octanoate); isoamyl alcohol	-	Enhances desirable volatile compounds in sequential fermentation	[11]

**Table 2:** Overview of methodologies for yeast isolation and characterization.

Method Category	Specific Method	Purpose	Outputs	Reference
Isolation	Serial Dilution Plating	Obtain pure yeast cultures from fruit matrices	Distinct colony morphologies on agar plates	[35]
	Macroscopic & Microscopic Observation	Initial identification and morphological assessment	Colony form, size, color, margin, elevation; cell shape, size	[8]
	Durham Tube Test	Assess fermentative capacity and CO_2_ production	Gas bubble formation and volume	[8]
Phenotypic Characterization	Physiological Tests (Sugar Fermentation, Temp, pH, Ethanol, SO_2_, NaCl Tolerance)	Evaluate metabolic capabilities and stress resilience	Growth/no growth under various conditions; sugar utilization patterns	[8]
	Enzymatic Activity & Metabolite Production (Acetic Acid, H_2_S, β-Glucosidase, Pectinase, Esterase, Protease, Lipase, Cellulolytic, Catalase)	Identify production of flavor/quality influencing compounds	Clear halos, color changes, bubble formation, precipitate formation	[8]
	DNA Extraction	Obtain genomic DNA for molecular analysis	Purified DNA sample	[35]
	Rep-PCR	Strain-level discrimination	Unique DNA banding patterns	[35]
	RFLP-PCR of 5.8S-ITS region	Species-level identification based on restriction patterns	Unique restriction fragment profiles	[35]
Molecular Identification	ITS Sequencing	Species-level identification	DNA sequence of ITS region	[37]
	26S rRNA Gene (D1/D2) Sequencing & Phylogenetic Analysis	Robust species identification and evolutionary relationships	Phylogenetic tree, sequence similarity to known species	[35]
	Phylogenomics & Functional Annotation	Decipher metabolic networks and functional potential	Identification of ‘fermentome’ and ‘flavorome’ genes	[31]
	Integrated Omics (Genomics, Proteomics, Metabolomics)	Comprehensive understanding of genetic and metabolic contributions	Identification of genes/enzymes for flavor, bioactive compounds	[38]
	Multi-stage Evaluation (Preliminary, Secondary, Final)	Optimize yeast strain selection for specific characteristics	Comprehensive assessment of fermentation performance, chemical, and sensory attributes	[39]
Advanced Screening	Response Surface Methodology (RSM)	Optimize fermentation conditions for specific compound production	Optimal parameters for desired metabolite yield (e.g., PEA)	[37]
	Sensory Analysis (Hedonic, CATA)	Evaluate consumer perception and quality	Sensory scores, descriptive terms	[37]
	Small-scale fermentation trials	Validate performance in realistic fruit musts	Fermentation vigor, CO_2_ release, improved wine composition (pH, ethanol, VOCs)	[8]

**Table 3:** Comparative effects of inoculation strategies on fruit beverage composition and sensory profile.

Strategy	Yeast Species	Fruit Matrix	Ethanol Production	Aroma/Flavor Compounds	Sensory Impact	Reference
	*S. bayanus*	Durian	5.3% *v*/*v*	Lower acetyl esters, higher alcohols; sulfur volatiles decline [44]	Less flavor complexity	[2]
	*T. delbrueckii*	Durian	5.8% *v*/*v*	Lower acetyl esters, higher alcohols; sulfur volatiles decline	Less flavor complexity	[2,44]
Single-Strain Inoculation	*S. cerevisiae* EC-1118	Red Dragon Fruit	8–9% *v*/*v*	High esters production	-	[10]
	*T. delbrueckii* Biodiva	Red Dragon Fruit	8–9% *v*/*v*	More higher alcohols, isoamyl acetate, terpenes; lower glycerol/acetic acid	Better antioxidant activity, color stability	[10]
	*L. thermotolerans*	Red Dragon Fruit	8–9% *v*/*v*	More lactic/succinic acids	Better antioxidant activity, color stability	[10]
	*S. bayanus* + *T. delbrueckii*	Durian	6.2% *v*/*v*	Higher acetyl esters, higher alcohols (isoamyl, 2-phenylethyl); new thioesters formed	Improved bouquet intensity & complexity	[44]
	*T. delbrueckii* Biodiva + *P. kluyveri*	Durian	Like *T. delbrueckii* monoculture	Similar ethyl esters, higher alcohols; moderately increased ethyl acetate [45]	More complex aromas	[45]
Co-fermentation	*T. delbrueckii* + *O. oeni*	Durian	6.06% *v*/*v*	Moderately increased higher alcohols, acetate esters, ethyl esters	Positive sensory impacts	[46]
	*S. cerevisiae* + *W. saturnus*	Mango	Like *S. cerevisiae* monoculture	Higher alcohols; reduced metallic/yeasty notes	Strong sulfury note retained, less unfavorable notes	[47]
	*T. delbrueckii* (75%) + *S. cerevisiae* (25%)	Moscato Branco	-	Lowest undesirable volatile fatty acids; highest 2-phenylethanol, acetates, ethyl esters	Highest general quality, aroma quality, flavor intensity (fruity, flowery)	[48]
	*T. delbrueckii* then *S. bayanus*	Durian	7.7% *v*/*v*	Higher acetyl esters, higher alcohols (isoamyl, 2-phenylethyl); new thioesters formed	Improved bouquet intensity & complexity	[44]
	*P. kluyveri* then *T. delbrueckii*	Durian	Similar to *T. delbrueckii* monoculture	Increased malic acid degradation, higher succinic acid; excessive ethyl acetate (≥80 mg/L)	-(potential for excessive ethyl acetate)	[45]
Sequential Inoculation	*W. saturnus* then *S. cerevisiae*	Mango	-	Higher desirable volatile compounds: ethyl esters (hexanoate, decanoate, octanoate), acetate esters (ethyl, isobutyl, isoamyl, 2-phenylethyl), isoamyl alcohol	More fruity and creamy notes	[11]
	*W. saturnus* then *S. cerevisiae*	Papaya	-	More acetate esters, fruitiness than simultaneous inoculation	More fruitiness	[49]

## Data Availability

No new data were created or analyzed in this study. Data sharing is not applicable to this article.
